# Prognostic value of *p16, p53*, and *pcna* in sarcoma and an evaluation of immune infiltration

**DOI:** 10.1186/s13018-022-03193-3

**Published:** 2022-06-10

**Authors:** Dechao Cai, Xiao Ma, Huihui Guo, Haotian Zhang, Ashuai Bian, Haoran Yu, Wendan Cheng

**Affiliations:** Department of Orthopedics, The Second Hospital of Anhui Medical University, 678 Furong Road, Hefei, 230601 China

**Keywords:** *p16* (*cdkn2a*) gene, *Tp53* gene, *Pcna* gene, Prognosis, Immune infiltration

## Abstract

**Background:**

*p16, p53,* and proliferating cell nuclear antigen *(pcna)* genes play significant roles in many chromatin modifications and have been found to be highly expressed in a variety of tumor tissues. Therefore, they have been used as target genes for some tumor therapies. However, the differential expressions of the *p16, p53,* and *pcna* genes in human sarcomas and their effects on prognosis have not been widely reported.

**Methods:**

The Oncomine dataset was used to analyze the transcription levels of *p16, p53*, and *pcna* genes, and the gene expression profile interactive analysis (GEPIA) dataset was used to analyze the differential expressions of *p16, p53*, and *pcna*. The expression levels of *p16, p53,* and *pcna* were further analyzed by Western Blotting. GEPIA and Kaplan–Meier analyses were used to analyze the prognostic value of *p16, p53*, and *pcna.* Furthermore, *p16, p53*, and *pcna* gene mutations and their association with overall survival (OS) and disease-free survival (DFS) were analyzed using cBioPortal datasets. In addition, genes co-expressed with *p16, p53,* and *pcna* were analyzed using Oncomine. The DAVID dataset was used to analyze the functional enrichment of *p16, p53, pcna,* and their co-expressed genes by Gene Ontology (GO) and Metascape were used to construct a network map. Finally, the immune cell infiltration of *p16, p53*, and *pcna* in patients with sarcoma was reported by Tumor Immune Estimation Resource (TIMER).

**Results:**

*p16, p53,* and *pcna* were up-regulated in human sarcoma tissues and almost all sarcoma cell lines. Western Blotting showed that the expression of *p16, p53,* and *pcna* was elevated in osteosarcoma cell lines. The expression of *pcna* was correlated with OS, the expression of *p16, p53,* and *pcna* was correlated with relapse-free survival, and the genetic mutation of *p16* was negatively correlated with OS and DFS. We also found that *p16, p53,* and *pcna* genes were positively/negatively correlated with immune cell infiltration in sarcoma.

**Conclusions:**

The results of this study showed that *p16, p53,* and *pcna* can significantly affect the survival and immune status of sarcoma patients. Therefore, *p16, p53,* and *pcna* could be used as potential biomarkers of prognosis and immune infiltration in human sarcoma and provide a possible therapeutic target for sarcoma.

## Introduction

Sarcoma is a rare group of heterogeneous tumors, mainly from the bone and soft tissue, and is highly invasive malignant tumors [1, 2]. Sarcomas can occur in almost all age groups; however, they are more common in adolescents and children and account for 10% of the malignant tumors in children and adolescents [3]. The exact etiopathogenesis of sarcomas is still unclear and needs further research. Osteosarcoma is one of the leading causes of cancer deaths in young people [4, 5]. Childhood and adolescents osteosarcoma has a high incidence [6, 7] and is reported at about 5% [8]. Osteosarcoma has a high rate of metastasis, and the effect of treatment and prognosis are poor [9]. Although surgery combined with chemotherapy slightly improves the survival rate of patients, there has been no substantial improvement in the survival rate in the past 40 years. In addition, the prognosis of patients with metastatic osteosarcoma is very poor, and the overall survival (OS) rate is 30% [10, 11].

Of the tumor suppressor genes, *p16* and *p53* are very important. Once inactivated, there is a proliferation of malignant cells. With a tumor suppressor gene protein product *cdkn2a*, *p16* mutations seriously affect the progression and prognosis of many tumors. *p53* is an important tumor suppressor gene [12, 13]. The *p53* gene mutation rate is very high. Due to changes in its spatial conformation, the *p53* gene loses the ability to regulate cell growth, cell apoptosis, and DNA repair, thus progressing from a tumor suppressor gene to a cancer gene. Proliferating cell nucleus antigen (*pcna*) plays an important role in DNA replication and repair. It forms a DNA containing homology trimer ring, anchoring along its side DNA polymerase and other editing enzymes that regulate the DNA and protein sequence motif of *pcna*—interacting protein tape (PIP—box), thereby influencing the development of tumor [14].

According to previous research, *p16*, *p53*, and *pcna* genes affect the development of various tumors in many ways. Ishida et al. reported, for example, that the lack of *p16* gene was associated with a 91% prevalence of esophageal squamous cell carcinoma and showed that the overexpression of the *p16* was caused by an imbalance in the RB1 pathway [15]. Wang and others reported the highest *p53* gene mutation frequency in liver cancer patients in China [16]. Ho et al. show that *pcna* was highly expressed in colon adenocarcinoma, with the expression of *pcna* in the distant metastasis of tumor as well [17]. However, the mechanisms of *p16*, *p53*, and *pcna* genes in sarcoma have not been widely reported.

The aim of this work was to study the expression of *p16*, *p53*, and *pcna* gene in sarcomas and evaluate its prognostic significance and association with immune cell infiltration.

## Results

### Transcription of *p16*, *p53*, and *pcna* in patients with sarcoma

**Fig. 1:**
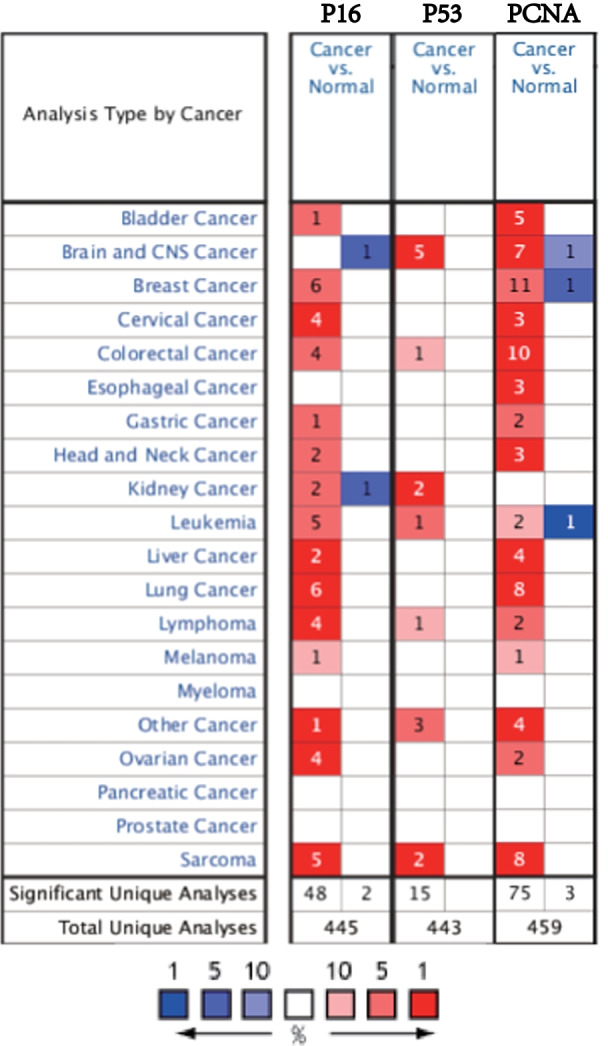
Expression levels of *p16, p53*, and *pcna* genes in different types of human cancer and normal samples

Based on Oncomine database data, we found high *p16*, *p53*, and *pcna* expressions in sarcoma (Fig. 1). In addition, the differential transcriptional expression levels of *p16, p53,* and *pcna* in sarcoma subtypes and corresponding normal tissues were analyzed using the Detwiller and Barretina sarcoma databases. The expression level of *p16* in the Detwiller's sarcoma database was up-regulated, and the multiple changes in these genes in leiomyosarcoma, pleomorphic liposarcoma, round cell liposarcoma, dedifferentiated liposarcoma, and malignant fibrous histiocytoma were 21.899, 25.731, 11.632, 4.897, and 4.789, respectively. Analysis of the Barretina sarcoma database showed that compared with normal tissue, multiple changes of *p16* expression levels in dedifferentiated liposarcoma, leiomyosarcoma, pleomorphic liposarcoma, myxoid fibrosarcoma, and myxoid/round cell liposarcoma were 6.870, 5.963, 6.395, 4.967, and 1.300, respectively.

According to the Detwiller sarcoma database analysis, multiple changes in *p53* expression in synovial sarcoma and fibrosarcoma compared with normal tissues were 2.660 and 1.885, respectively. In the Barretina sarcoma database, multiple changes in *p53* expression in myxoid/round cell liposarcoma and dedifferentiated liposarcoma compared with normal tissues were 4.235 and 1.755, respectively.

Based on the Detwiller sarcoma database analysis, the multiple changes in *pcna* expression in pleomorphic liposarcoma, leiomyosarcoma, fibrosarcoma, synovial sarcoma, and malignant fibrous histiocytoma were 3.836, 3.155, 3.750, 2.385, and 3.444, respectively. In the Barretina sarcoma database, the multiple changes of *pcna* expression in pleomorphic liposarcoma, leiomyosarcoma, myxofibrosarcoma, dedifferentiated liposarcoma, and myxoid/round cell liposarcoma were 4.323, 5.521, 4.905, 2.874, and 2.492, respectively.

### mRNA expression of *p16, p53,* and *pcna* in sarcoma

**Fig. 2:**
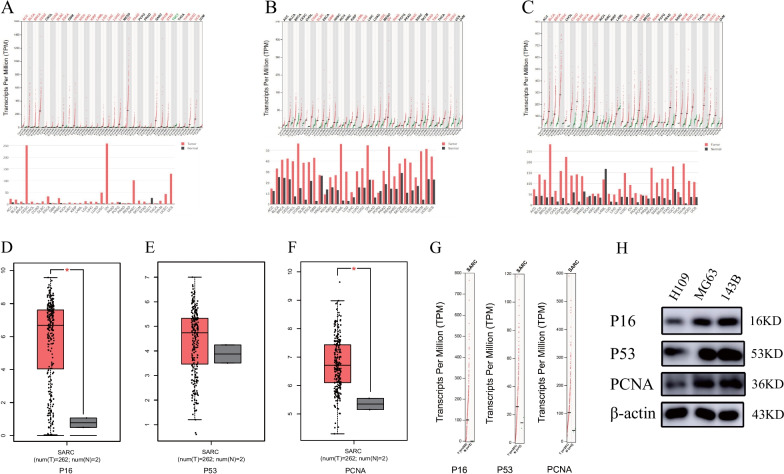
Expression levels of *p16, p53*, and *pcna* genes in sarcomas. **A**–**C** The expression levels of *p16, p53*, and *pcna* in generalized carcinoma, and **D**–**G** the expression levels of *p16, p53*, and *pcna* in sarcoma. Each point represents a separate sample. **H **Expression of *p16, p53,* and *pcna* in normal osteoblasts and two osteosarcoma cell lines by Western Blotting. *P < 0.05

Using the GEPIA tool, we found that the *p16* and *pcna* mRNA levels were significantly raised in sarcoma compared to normal tissue (*P* > 0.05), although the level of mRNA expression of *p53* showed no significant increase (Fig. 2A–G).

To further evaluate the expression of *p16, p53,* and *pcna* in osteosarcoma, we selected two osteosarcoma cell lines to detect their protein expression. Normal osteoblasts constituted the control group. Western Blotting results showed that the expression of *p16, p53,* and *pcna* in various osteosarcoma cell lines was significantly higher than that in normal osteoblasts (Fig. 2H), consistent with the results of the Oncomine and GEPIA database analysis.

### Prognostic value of *p16*, *p53*, and *pcna* gene in sarcoma patients

**Fig. 3:**
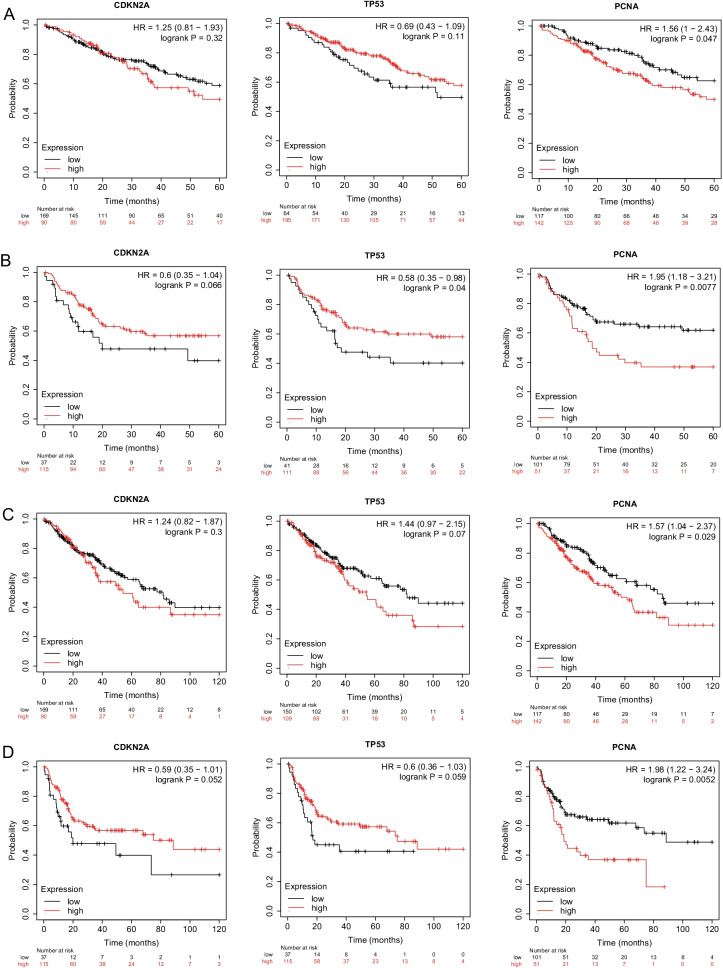
K-M survival curve. **A** Association between high expression levels of *p16*, *p53*, and *pcna* genes and 5-year overall survival in patients with sarcoma. **B** Association between elevated levels of *p16*, *p53*, and *pcna* gene expression and 5-year relapse-free survival (RFS) in patients with sarcoma. **C** Association between high expression levels of *p16*, *p53*, and *pcna* genes and 10-year overall survival in patients with sarcoma. **D** Association between elevated levels of *p16*, *p53*, and *pcna* gene expression and 10-year relapse-free survival in patients with sarcoma

**Fig. 4:**
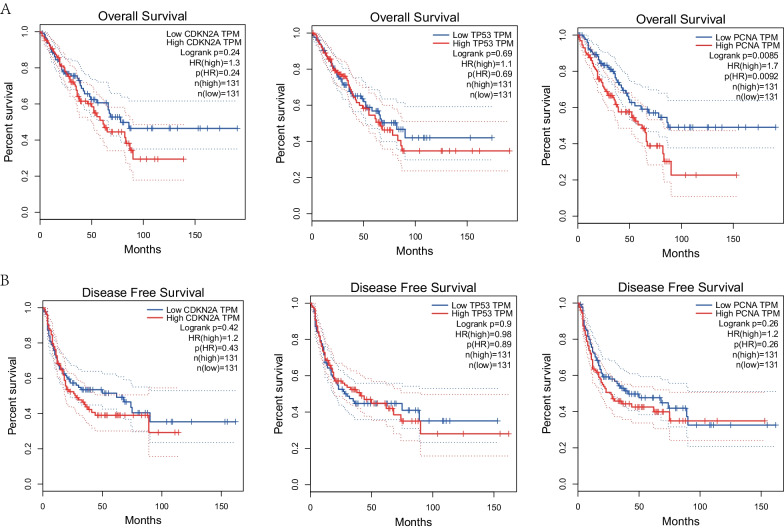
GEPIA survival curve. **A** Association between elevated *p16*, *p53*, and *pcna* gene expression and overall survival in patients with sarcoma. **B** Association between expression levels of *p16*, *p53*, and *pcna* genes and disease-free survival (DFS) in patients with sarcoma

Kaplan–Meier plots and GEPIA evaluation demonstrated the prognostic effect of *p16*, *p53*, and *pcna* gene expression in sarcoma patients. The expression of *pcna* was related to a poor 5- and 10-year OS. With the increase in *p53* expression, the 5-year recurrence-free survival (RFS) was up-regulated. The increase in *pcna* expression was associated with a lowering of the 5- and 10-year RFS in sarcoma, and in recurrent sarcomas, *pcna* was found to be differentially expressed (Fig. 3). *p16* tended to affect the RFS in sarcoma, but this was not statistically significant. We found an inverse correlation between *pcna* expression and the OS in sarcoma patients. The expression of *p16*, *p53*, and *pcna* was not related to DFS in patients with sarcoma (Fig. 4).

### Mutations in *p16*, *p53*, and *pcna* genes and their effects on OS and DFS in sarcoma patients

**Fig. 5:**
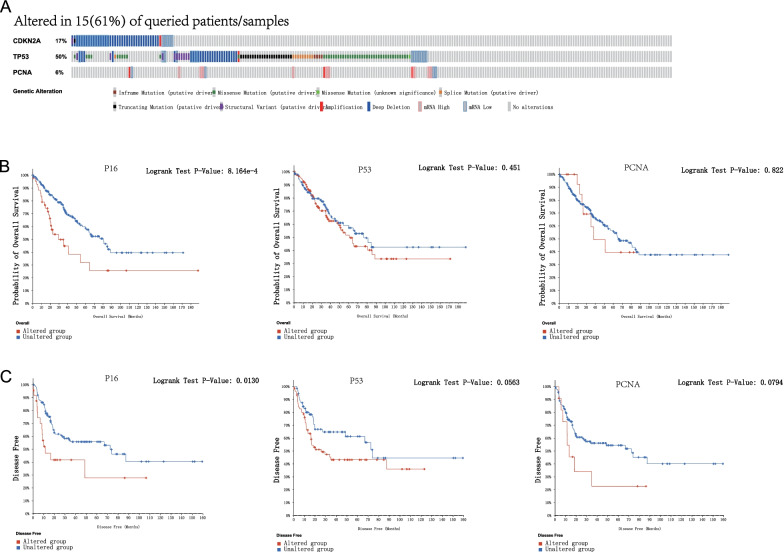
Gene mutations in *p16*, *p53*, and *pcna* and their association with OS and DFS in patients with sarcoma (cBioPortal). High mutation rates of *p16*, *p53*, and *pcna* (61%) were observed in SARC patients, with mutation rates of 17, 50, and 6%, respectively (**A**). Relationship between genetic changes of *p16*, *p53,* and *pcna* and OS (**B**) and DFS (**C**) in SARC patients

Next, we analyzed the mutations of *p16*, *p53*, and *pcna* genes and its influence on OS and DFS in sarcoma. High mutation rates of *p16*, *p53*, and *pcna* were observed in sarcomas; 154 of the 254 sarcomas sequenced showed gene mutations with a mutation rate of 61%. Of the three, *p16* and *p53* gene mutation rates were relatively high, at 17 and 50%, respectively. *pcna* gene mutation rate was 6% (Fig. 5A). In addition, the results of Kaplan–Meier survival analysis showed that genetic mutations in *p16* represented a decrease in OS (Fig. 5B, *P* = 8.164E − 4) and DFS (Fig. 5C, *P* = 0.0130) in sarcoma patients. The high frequency of *p53* gene mutations tends to be negatively correlated with DFS in sarcoma, but it is not significant (*P* = 0.0563). The *pcna* gene mutations were not shown to significantly affect the OS and DFS in sarcoma patients. These results suggest that *p16* and *p53* gene mutations may significantly influence the outcome of patients with sarcoma.

### Identification of genes co-expressed with *p16*, *p53*, and *pcna* genes in osteosarcoma

**Fig. 6:**
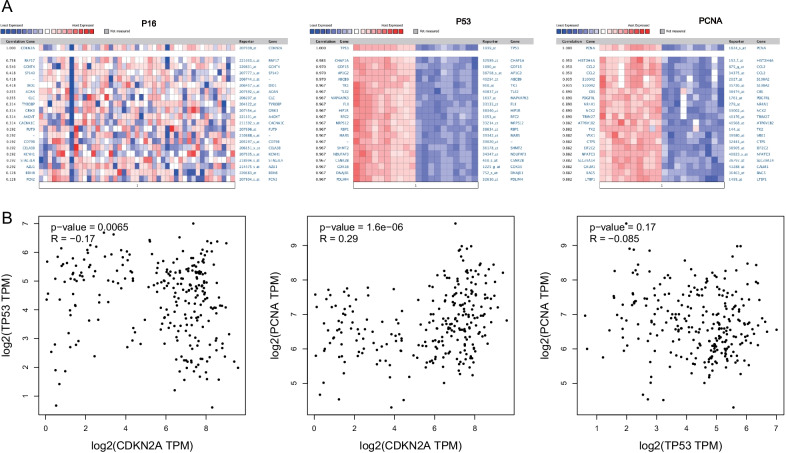
**A** Genes co-expressed with *p16*, *p53* and *pcna* genes in osteosarcoma. **B** Correlation between *p16*, *p53,* and *pcna* genes in sarcomas

We used the Oncomine dataset to detect genes co-expressed with *p16*, *p53*, and *pcna* in osteosarcoma. The genes co-expressed with *p16* were *rnf17, gcnt4, sp140, dio1, acan, clc, tyrobp, grik3, a4gnt,* and *cacna1c*. The genes co-expressed with *p53* were *chaf1a, gdf15, ap1g2, abcb9, tk1, tle2, mapkapk3, fli1, hip1r,* and *rfc2*. The genes co-expressed with *pcna* were *hist2h4a, ccl2, s100a2, cbs, pdgfrl, nr4a1, nck2, trim27, atp6v1b2,* and *tk2* (Fig. 6A).

Then, we analyzed the co-expression between *p16*, *p53*, and the *pcna* gene. The results showed that *p16* and *pcna* were positively correlated (*R* = 0.29, *P* < 0.05), *p53*, and *p16* showed negative correlation (*R* = 0.17, *P* < 0.05). There was no significant correlation between *p53* and *pcna* (Fig. 6B).

**Fig. 7:**
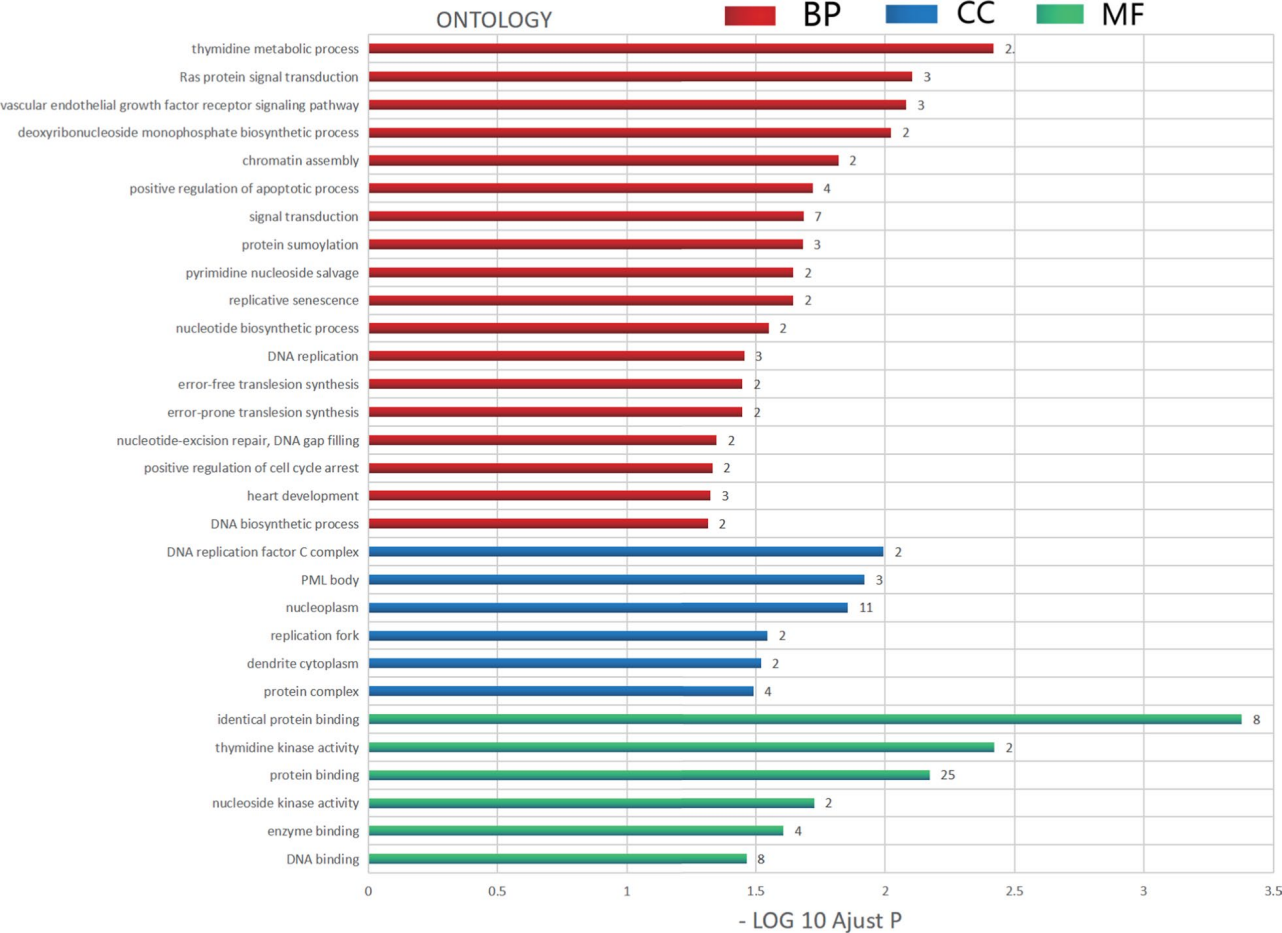
GO analysis of *p16, p53*, and *pcna* genes and co-expressed genes

Next, the enrichment of David analysis showed that *p16*, *p53*, and *pcna* and co-expressed genes mainly involved were: GO:0,046,104 (thymidine metabolic process), GO:0,007,265 (Ras protein signal transduction), GO:0,048,010 (vascular endothelial growth factor receptor signaling pathway), GO:0,009,157 (deoxyribonucleoside monophosphate biosynthetic process), and GO:0,031,497 (chromatin assembly) of biological processes. Cellular components (CCs), GO:0,005,663 (DNA replication factor C complex), GO:0,016,605 (promyelocytic leukemia [PML] body), GO:0,005,654 (nucleoplasm), GO:0,005,657 (replication fork), and GO:0,032,839 (dendrite cytoplasm) were enriched, and molecular functions (MFs): GO:0,042,802 (the same protein binding): GO:0,004,797 (thymidine kinase activity): GO:0,005,515 (protein binding): GO:0,019,206 (nucleoside kinase activity), GO: 0,019,899 (binding) were enriched (Fig. 7 and Table 1).

**Table 1:** GO analysis of *p16, p53, pcna*, and co-expressed genes

The enriched	GO-ID	Description	Count	*P* Value	Genes
BP	0,046,104	Thymidine metabolic process	2	0.00380782	*tk2, tk1*
BP	0,007,265	Ras protein signal transduction	3	0.007837465	*cdkn2a, tp53, mapkapk3*
BP	0,048,010	Vascular endothelial growth factor receptor signaling pathway	3	0.00827545	*nck2, ccl2, mapkapk3*
BP	0,009,157	Deoxyribonucleoside monophosphate biosynthetic process	2	0.009493227	*tk2, tk1*
BP	0,031,497	Chromatin assembly	2	0.015147194	*tp53, chaf1a*
BP	0,043,065	Positive regulation of apoptotic process	4	0.019103663	*hip1r, cdkn2a, tp53, nr4a1*
BP	0,007,165	Signal transduction	7	0.020711051	*nck2, tle2, tyrobp, ccl2, gdf15, mapkapk3, nr4a1*
BP	0,016,925	Protein sumoylation	3	0.020834415	*cdkn2a, pcna, tp53*
BP	0,090,399	Replicative senescence	2	0.022637202	*cdkn2a, tp53*
BP	0,043,097	Pyrimidine nucleoside salvage	2	0.022637202	*tk2, tk1*
BP	0,009,165	Nucleotide biosynthetic process	2	0.028218465	*tk2, tk1*
BP	0,006,260	DNA replication	3	0.035043323	*pcna, rfc2, chaf1a*
BP	0,042,276	Error-prone translesion synthesis	2	0.035612139	*pcna, rfc2*
BP	0,070,987	Error-free translesion synthesis	2	0.035612139	*pcna, rfc2*
BP	0,006,297	Nucleotide-excision repair, DNA gap filling	2	0.044777623	*pcna, rfc2*
BP	0,071,158	Positive regulation of cell cycle arrest	2	0.046600567	*cdkn2a, tp53*
BP	0,007,507	Heart development	3	0.047320182	*acan, pcna, cacna1c*
BP	0,071,897	DNA biosynthetic process	2	0.04842014	*tk2, tk1*
CC	0,005,663	DNA replication factor C complex	2	0.010164404	*pcna, rfc2*
CC	0,016,605	PML body	3	0.012026098	*tp53, sp140, trim27*
CC	0,005,654	Nucleoplasm	11	0.013931808	*cdkn2a, tle2, flii, pcna, tp53, rfc2, sp140, trim27, mapkapk3, nr4a1, hist2h4a*
CC	0,005,657	Replication fork	2	0.028540071	*pcna, tp53*
CC	0,032,839	Dendrite cytoplasm	2	0.030194119	*hip1r, grik3*
CC	0,043,234	Protein complex	4	0.032294565	*cdkn2a, tp53, chaf1a, hist2h4a*
MF	0,042,802	Identical protein binding	8	4.19E-04	*s100a2, pcna, tp53, cbs, tyrobp, trim27, chaf1a, tk1*
MF	0,004,797	Thymidine kinase activity	2	0.003787763	*tk2, tk1*
MF	0,005,515	Protein binding	25	0.006734124	*s100a2, hip1r, tle2, rfc2, sp140, nr4a1, tk1, hist2h4a, acan, cacna1c, clc, cbs, gdf15, ap1g2, chaf1a, nck2, pcna, tp53, abcb9, cdkn2a, flii, tyrobp, atp6v1b2, trim27, mapkapk3*
MF	0,019,206	Nucleoside kinase activity	2	0.018800305	*tk2, tk1*
MF	0,019,899	Enzyme binding	4	0.024733293	*pcna, brox, gabrd, iqch*
MF	0,003,677	DNA binding	8	0.034312324	*cdkn2a, pcna, tp53,rfc2, sp140, trim27, neu2, hist2h4a*

**Fig. 8:**
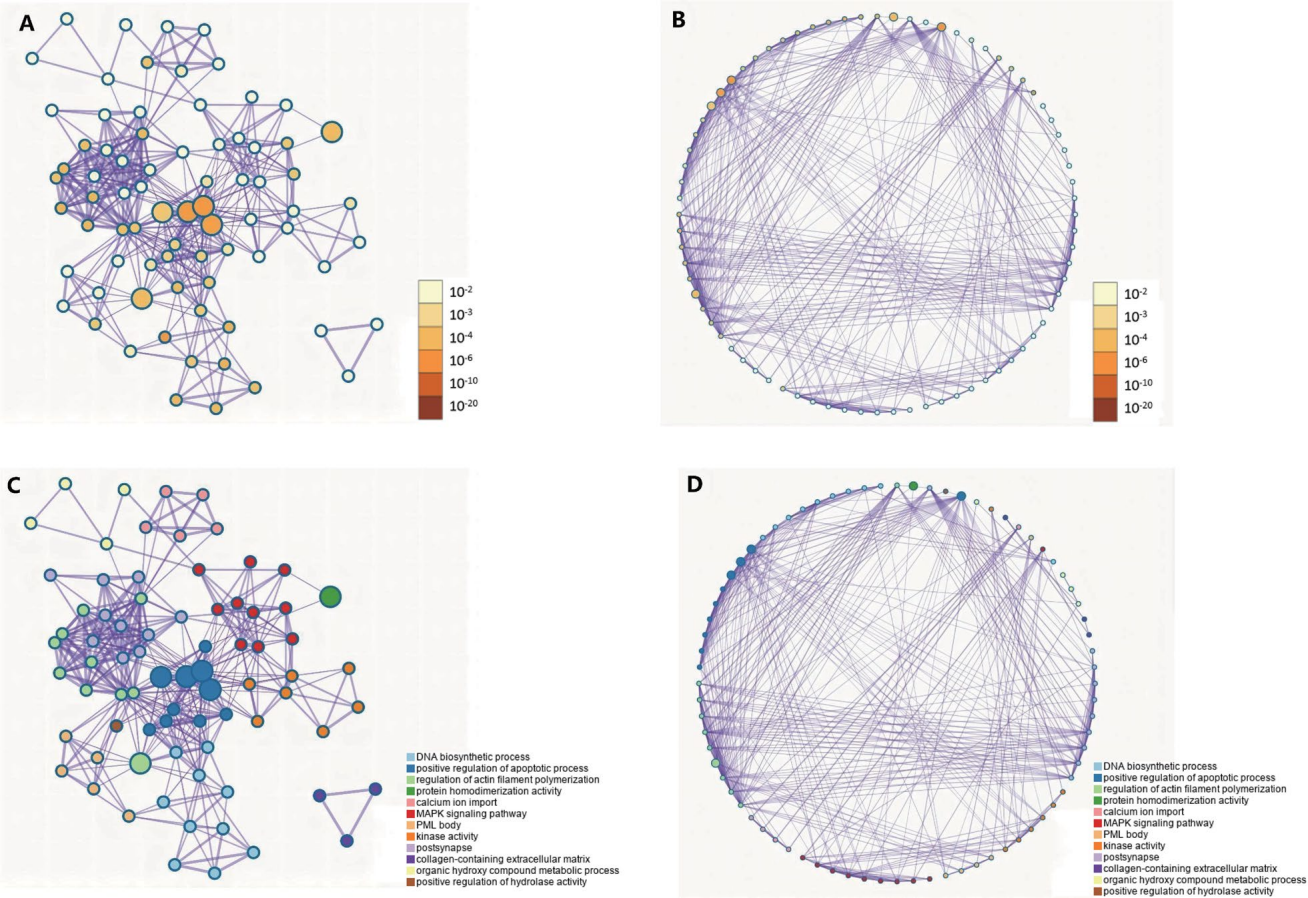
**A** and **B** are colored by p values, where items containing more genes have more significant *p* values, and **C** and **D** are colored by cluster IDS, where nodes sharing the same cluster ID are usually close to each other

To intuitively show the relationship between the enrichment of each gene (*p16*, *p53*, and *pcna* and their co-expressed genes), we used Metascape to construct a network diagram, the cytoscape visualization network with each node representing a rich group (Fig. 8).

### Expression of *p16*, *p53*, and *pcna* genes and degree of immune infiltration in sarcoma

**Fig. 9:**
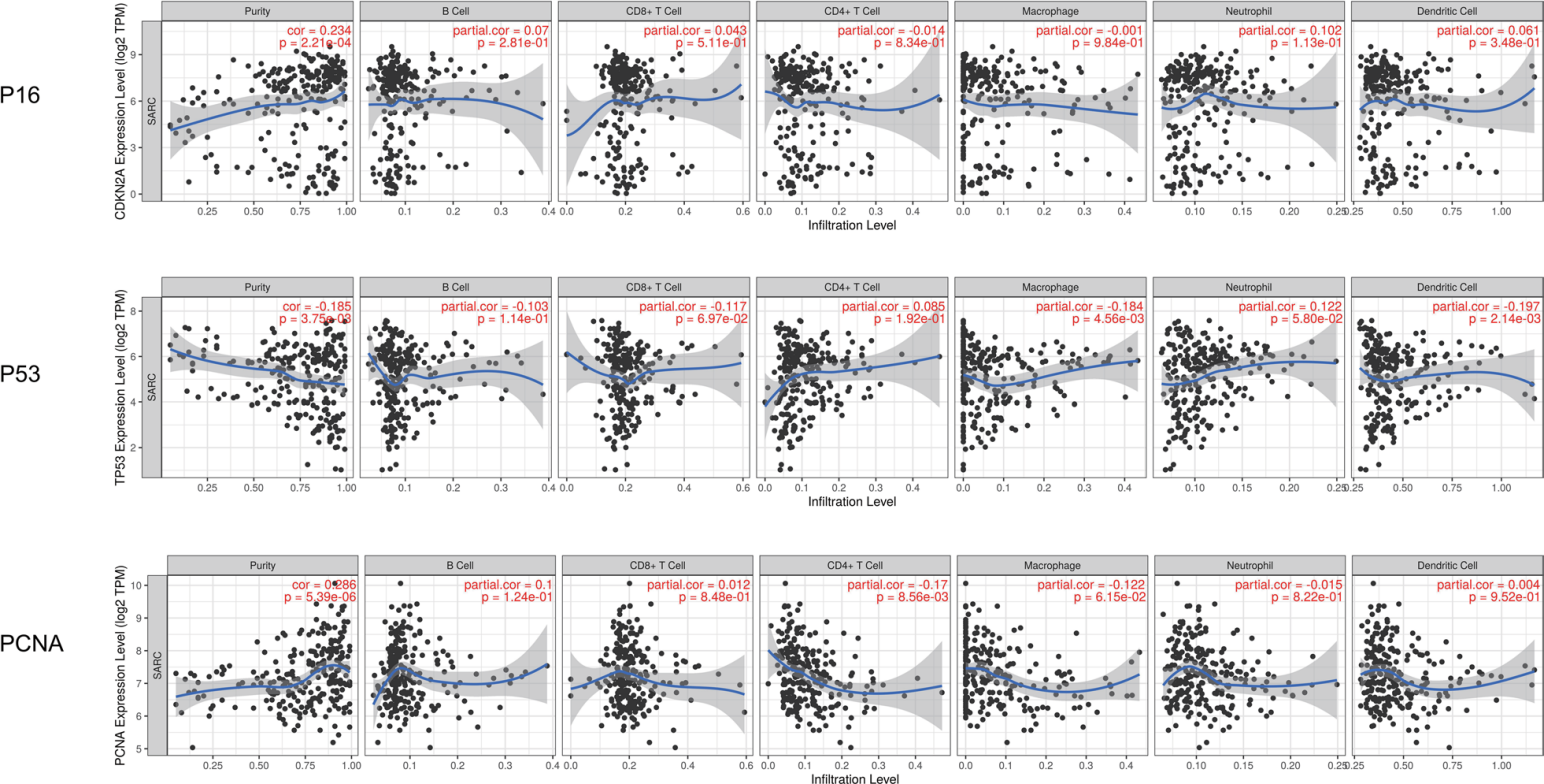
Relationship between differential expression of *p16*, *p53*, and *pcna* genes and immune cell infiltration

In this study, the TIMER was used to explore the correlation between the expression of *p16*, *p53*, and *pcna* genes and immune cell infiltration within the sarcoma. TIMER analysis showed that *p16* and infiltrating neutrophils showed a significant correlation (*R* = 0.102, *P* < 0.05). Similarly, the expression of *p53* and B lymphocyte infiltration also showed a significant correlation (*R* = 0.103, *P* < 0.05), and so did CD8 + T cells (*R* = 0.117, *P* < 0.05), macrophages (*R* = 0.184, *P* < 0.05), neutrophils (R = 0.122, *P* < 0.05), and dendritic cells (*R* = 0.197, *P* < 0.05). The expression of *pcna* and B lymphocyte infiltration showed a significant correlation (*R* = 0.1, *P* < 0.05), so did CD4 + T cells (*R* = 0.17, *P* < 0.05), and macrophages (*R* = 0.122, *P* < 0.05) (Fig. 9).

## Discussion

*p16*, *p53*, and *pcna* genes have been shown to play an irreplaceable role in the occurrence and development of a variety of malignant tumors. However, gene differential expression of *p16*, *p53*, and *pcna* in sarcoma and its influence on prognosis have not been widely reported. At present, tumor immunotherapy has become a research hotspot, and reports have said that effective immunotherapy has been carried out for many different types of malignant tumors [18–22]. In addition, clinical studies have made some progress in detecting personalized new vaccine antigens in patients with tumors [23]. There is growing attention toward immune-related research in sarcomas and evaluation of immunotherapy combined with radiotherapy, chemotherapy, and some targeted treatment. We conducted this study to reveal the relationship between *p16*, *p53*, and *pcna* genes in human sarcomas with patient prognosis and immune infiltration.

The *p16* gene is a very important anticancer gene. Once inactivated, malignant proliferation of cells occurs. At present, the lack of homozygous, nonsense, missense, and *p16* frameshift mutations was found in many types of tumor tissue, showing that *p16* gene deletions and mutations are widely involved in tumor formation. Zeng et al. [24] reported a method to monitor the direct effects of the genetic damage of *p16* on low-channel primary human melanocytes using precision engineering and digital holography. They observed that the deletion of *p16* promotes melanocyte movement through transcriptional activation of *brn2*, as well as increases the invading and metastasizing ability of the melanoma cells, thereby showing that *p16* deletion leads to melanoma progression. Sarun et al. [25] reported that homozygous deletion of *cdkn2a* is one of the most common gene mutations in malignant pleural mesothelioma. Tirode et al. [26] reported that *p16* gene mutation frequency of Ewing's sarcoma was 12%. Campbell et al. [27] reported the important role of *cdkn2a* (*p16*) gene in the etiology of pancreatic cancer and thought it to be one of the most common mutations in pancreatic cancer. They also found that *cdkn2a* promoter methylation plays a key role in the pathogenesis and prognosis of pancreatic cancer [36]. Previous research has shown that changes in the *p16* gene can significantly affect the prognosis of patients with many types of tumors. Botana-Rial et al. [28] reported that patients with *p16 ink4a*/methylation have a shorter survival time than those without methylation. Pessôan et al. [29] reported that *p16* has a high mutation rate in gliomas, and the higher the degree of malignancy of gliomas, the higher the frequency of *p16* mutation (16.7%) and the worse the prognosis. Trietsch and others reported that *p16* was often mutated in human papilloma virus-negative vulvar squamous cell carcinoma, and the 5-year survival rate of patients with obvious mutations was worse than patients without mutations [30]. El-mokadem et al. [31] found that when patients with renal clear cell carcinoma developed *p16* gene mutation, the degree of malignancy was usually high, and *p16* gene mutation increased the risk of tumor metastasis, leading to higher tumor recurrence and metastasis, which was associated with poor prognosis. These results are similar to the results of our study. The genetic variation rate of *p16* is more common in sarcoma patients (17%), and genetic mutations of *p16* are associated with poor OS and DFS. It is reported that overexpression of *p16* in traditional esophageal squamous cell carcinoma is associated with a better clinical prognosis [32, 33]. Zhou et al. [34] found that *p16* positive lung adenocarcinoma had a good prognosis. Kommoss et al. [35] found that *p16*-negative status was an indicator of poor prognosis in clear cell ovarian cancer and mucinous ovarian cancer subgroups. Barber et al. [36] found that in oropharyngeal squamous cell carcinoma, up-regulation of *p16* expression implied better RFS. In the current study, the up-regulation of *p16* expression was found to increase the RFS of sarcoma patients, but this was not statistically significant (*P* = 0.052). There was no significant correlation between the expression of *p16* and OS in patients.

As a transcription factor, *p53* suppresses cancer mainly through selective transcriptional regulation of multiple target genes, including regulation of apoptosis, cell cycle arrest, senescence, DNA repair, and metabolism [37–39]. The existing research is the same as the results of this work; the mutation rate of the *p53* gene is so high that it occurs in more than 50% of human malignant tumors [40–42]. In many human tumors, *p53* plays an important role in tumorigenesis and progression through gene mutation and other mechanisms, including the amplification and/or overexpression of *p53*-negative regulators, such as *mdm2* and *mdm4* [43]. It has been reported that mutation or downregulation of *p53* (encoded by *tp53*) accelerates the occurrence and malignant progression of esophageal squamous cell carcinoma [44]. Hou et al. [45] found that the Claudin 7 closed protein gene (*CLDN7*) located downstream of *p53* on the short arm of chromosome 17 is regulated by *WTp53* by binding to the promoter region of colorectal cancer. Once *p53* mutation or deletion occurs, the tumor inhibitory function of *CLDN7* is lost, indicating that the tumor inhibitory effect of *CLDN7* in colorectal cancer is closely related to the status of *p53*. Rodrigues et al. [46] have shown that 80% of patients with mantle cell lymphoma with *tp53* mutations die within the first 5 years of diagnosis. Marcus and Ladds proposed the use of dihydrowhey acid dehydrogenase and *p53* activation as tumor treatment targets, thus effectively killing tumor cells [47]. Takamatsu et al. proposed that inhibiting *p53* aggregation through various methods, that is, reducing the evolutionary ability of *p53* to nuclear stress, may enhance the effectiveness of cancer treatment and reduce cancer recurrence [48]. George et al. [49] reported that the mutation of the tumor suppressor gene *tp53* is associated with poor survival of patients with acute myeloid leukemia and proposed a new molecular targeted therapy with the main goal of degrading or inactivating mutant *p53* or restoring *WTp53* to restore normal *tp53* function. Saleh et al. [50] reported that *p53* gene mutations are very frequent in high-grade serous ovarian cancer (HGSC), while the frequency of *p53* mutations in normal organs is relatively low, making *p53* an attractive target for specific therapy of HGSC. These results were similar to ours; the high-frequency genetic changes in *p53* were associated with poor DFS in sarcoma patients, but this was not statistically significant (*P* = 0.0563).

As a hub protein, *pcna* is considered to be a key regulator of DNA and cell cycle regulation. The expression of *pcna* is found to be up-regulated in many tumor types, and its overexpression is thought to be related to cancer virulence [51]. Tumor cells have strong proliferative activity and can be used as an index to evaluate the state of cell proliferation. Therefore, *pcna* has been studied in many tumors, involving the relationship between *pcna* and tumor occurrence, development, prognosis, recurrence and metastasis, tumor markers, and so on. It is known that high expressions of *pcna* can promote the proliferation of lung cancer cells and the ability to invade adjacent tissues; therefore, it can be used as a new molecular targeted marker for the diagnosis and treatment of lung cancer [52–54]. Jin et al. [55] have shown that excessive up-regulation of *pcna* expression is one of the factors that can directly affect the prognosis of rectal cancer, which significantly reduces the survival rate of patients. Wang et al. [56] found that the transforming growth factor (TGF)-β1 signaling pathway may affect cell growth, cell cycle distribution, and apoptosis by regulating the expression of *pcna* and other molecules in hepatocellular carcinoma. Hu et al. [57] reported that *pcna* expression in gastric cancer tissues was significantly up-regulated compared with normal tissues, and the positive expression of *pcna* is a risk factor for the prognosis of patients with gastric cancer. Qin et al. [58] reported that in glioblastoma (GBM), a type of microRNA (miR-1258), inhibits *pcna* transcription by directly targeting *e2f1*, which provides a new potential target for GBM therapy and other *e2f1*-driven cancers. According to the above reports, the up-regulated expression of *pcna* is related to the poor prognosis of patients with tumors, which is similar to the results we analyzed using Kaplan–Meier graphs, indicating that the increased expression of *pcna* is related to poor 5- and 10-year OS and RFS in sarcoma patients. Thus, the up-regulation of *pcna* expression means that patients with sarcoma are more likely to relapse.

A growing body of evidence indicates that the tumor microenvironment plays an important role in the regulation of the proliferation and metastasis [59, 60]. A variety of immune cells in the tumor microenvironment can promote or inhibit the activity of tumor cells. The tumor microenvironment is thought to be an important determinant of the clinical prognosis and response to immunotherapy [61, 62]. Adib et al. [63] found that patients with *cdkn2a* -altered urothelial cancer had decreased *pd-l1* expression in tumor-infiltrating immune cells, as well as decreased T cell receptors, antigen processing, and activation of interferon-gamma pathways. In urothelial carcinoma, the high expression of *pd-l1* in tumor-infiltrating immune cells is associated with an enhanced efficacy of immune checkpoint inhibitors and a good prognosis. After systematically exploring the differentially expressed genes related to immunity in hepatocellular carcinoma, Luo et al. [64] concluded that the high expression of *cdkn2a* is associated with a poor prognosis of hepatocellular carcinoma and a decrease in immune infiltration. The up-regulation of *cdkn2a* expression in hepatocellular carcinoma may be related to the involvement of *cdkn2a* in the MAPK signaling pathway and the diversity of hepatocellular carcinoma. The degree of tumor immune infiltration will lead to the destruction of the immune microenvironment and immune escape. Tan et al. have proved that the new Golgi programming caused by *p53* mutation is an important driver of cancer over secretion. The mechanism is as follows: *p53* deletion increases the expression of the Golgi scaffold protein, progesterone, and fat receptor 11 (PAQR11). The secretion of PAQR11-dependent protease PLAU can stimulate the autocrine of PLAU receptor/signal transduction and transcriptional 3-dependent pathway activator, which up-regulates the expression of PAQR11, thus completing a feedforward cycle that amplifies the secretion of premetastatic proteins. Blocking the secretion of PAQR11 can improve the immunosuppressive process in the tumor microenvironment [65]. Kurie et al. [66] and Tan et al. found that *tp53* deletion alleviates G55 (stacking protein 55 kDa) and myosin IIA activated G55-dependent secretion from miR-34a-dependent silencing. The G55-dependent secretory protein enhances the proliferation and invasion of *tp53*-deficient LUAD cells and the angiogenesis and CD8 + T cell depletion in the tumor microenvironment. Wang et al. [67] found that inhibiting the *Y211* phosphorylation of *pcna* causes the failure of the replication fork, which drives the biosynthesis of cellular solute ssDNA (single-stranded DNA) into cellular solute. It is suggested that the progress of *pcna Y211* phosphorylation in nuclear DNA replication is related to the DNA sensing cascade initiated by cGAS (cyclic GMP-AMP synthase) in the cytoplasm, thus regulating immune surveillance to inhibit tumor metastasis. Understanding the tumor microenvironment may contribute to a better understanding of tumor immune cell interactions to predict response to immunotherapy [68]. In this work, we found that *p16*, *p53*, and *pcna* can be used as surrogate immune markers for sarcoma.

This work had the following limitations: First of all, the data were analyzed from an online database, and there was a lack of adequate in vivo and in vitro testing. Second, the sample size was small. Therefore, a follow-up study with larger sample size is required.

## Conclusion

In short, our research suggests that *p16*, *p53*, and *pcna* are overexpressed in human sarcomas. The expression of *pcna* was correlated with OS, the expression of *p16*, *p53*, and *pcna* was correlated with RFS, and genetic mutations in *p16* were negatively correlated with OS and DFS. The *p16, p53,* and *pcna* genes were positively/negatively correlated with immune cell infiltration in sarcoma. These results suggest that the *p16, p53* and *pcna* genes significantly affect the prognosis of human sarcomas and are of vital value in immunotherapy. Our work may provide a reference for the choice of new prognostic biomarkers and tumor immunity, and the goal of treatment of sarcomas, providing directions for further research.

## Materials and methods

### Oncomine analysis

Oncomine database (https://www.oncomine.org/resource/login.html) is usually used to analyze the DNA or RNA sequence of cancer, and genome-wide expression data from malignant tumors [69]. In this work, Oncomine transcription was used to explore the differential expression of *p16, p53*, and *pcna* in various cancer tissues.

### Gene expression patterns of interaction analysis (GEPIA) assessment of datasets

GEPIA datasets (http://gepia.cancer-pku.cn/) are often used to analyze data generated by the cancer genome atlas project [70]. In this work, we use the GEPIA to explore the differential expression of *p16, p53*, and *pcna* genes.

### Reagents and cell lines

Human osteosarcoma cell lines MG63 and 143B were purchased from the Shanghai Institute of Cell Biology, Chinese Academy of Sciences. Human primary osteoblasts (Cat.No.GN-H109) were obtained from the Gaining Biological Company (Shanghai, China). The cells were cultured in DMEM medium supplemented with 10% FBS (fetal bovine serum), streptomycin, and penicillin at 37 °C and 5% CO_2_. Anti-p16 antibody (item no. AF5484), anti-p53 antibody (item no. AF0865), anti-PCNA antibody (item no. AF0239), and anti-β-actin antibody (item no. AF7018) were purchased from Affinity Biosciences (Jiangsu, China).

### Protein extraction and Western Blotting test

Total proteins were isolated from cells using the RIPA buffer (Solarbio). The Bradford assay (Bio-Rad Laboratories) was used to quantify protein. The protein was separated on 10% sodium dodecyl sulfate–polyacrylamide gel (SDS-PAGE) and then transferred to a polyvinylidene fluoride (PVDF) membrane (Millipore, USA). The resulting PVDF film was immersed in 5% skim milk and sealed for about 1 h. Next, the membrane was incubated overnight with anti-p16 (1VO1000, Affinity, China), anti-p53 (1VO1000, Affinity, China), and anti-PCNA (1REO1000, Affinity, China) antibodies at 4 °C and incubated with the corresponding secondary antibody (rabbit) at room temperature for 2 h. Finally, autoradiography was performed with the ECL kit (Thermo Fisher Scientific).

### Survival analysis

We use the Kaplan–Meier plotter database (https://kmplot.com/analysis/index.php? *P* = service&cancer = pancancer_rna seq) to investigate the effects of *p16, p53*, and *pcna* expression on the survival in sarcoma patients [71, 72]. This was compared with the survival curve obtained by the GEPIA dataset analysis.

### CBioPortal data analysis

In this work, cBioPortal (www.cbioportal.org) was used to analyze the gene mutations of *p16, p53,* and *pcna* and study their effect on OS and disease-free survival (DFS) in sarcoma patients [73].

### Enrichment analysis of co-expressed genes

David database (http://david.ncifcrf.gov) provides a comprehensive set of functional annotation tool for researchers to understand the biological significance behind a string of genes [74]. It was used in this study to perform ontology (go) analysis of enrichment of *p16, p53*, and *pcna*, and the genes. Metascape (https://metascape.org) has been used to provide a comprehensive analysis of the gene annotation and resources, integrating a variety of functions, such as enrichment, interactive group analysis, gene function annotation and members of the search, and providing outputs as unique visual graphics [75]. In this work, we used Metascape to build a network graph of related genes.

### Tumor immune to assess resource (TIMER) analysis

Timer web server (https://cistrome.shinyapps.io/timer/) is a comprehensive resource system that analyzes immune cell infiltration across different types of cancer [76, 77]. In this work, the TIMER was used to study the relationship between the expression of *p16, p53*, and *pcna* and immune cells penetrating the sarcoma.

## Data Availability

The datasets generated and/or analyzed during the current study are available in the TCGA repository, [https://www.tcga.org/].

## Abbreviations

BP: Biological process
CC: Cellular component
CCC: Clear cell ovarian cancer
DAVID: Database for annotation, visualization, and integrated discovery
DFS: Disease-free survival
GBM: Glioblastoma
GEPIA: Gene expression profile interactive analysis
GO: Gene Ontology
HGSC: High-grade serous ovarian cancer
MC: Mucinous ovarian cancer
MF: Molecular function
OS: Overall survival
*pcna*: Proliferating cell nuclear antigen
PML: Promyelocytic leukemia
RFS: Recurrence-free survival
DFS: Disease-free survival
TCGA: The cancer genome atlas
TIMER: Tumor immune estimation resource
FBS: Fetal bovine serum
SDS-PAGE: Sodium dodecyl sulfate–polyacrylamide gel
PVDF: Polyvinylidene fluoride
